# *Faecalibacterium prausnitzii* Is Associated with Disease Severity in MASLD but Its Supplementation Does Not Improve Diet-Induced Steatohepatitis in Mice

**DOI:** 10.3390/microorganisms13030675

**Published:** 2025-03-18

**Authors:** Eliane Münte, Greta Viebahn, Amit Khurana, Jumpei Fujiki, Tomohiro Nakamura, Sonja Lang, Münevver Demir, Bernd Schnabl, Phillipp Hartmann

**Affiliations:** 1 Department of Pediatrics, University of California San Diego, La Jolla, CA 92093, USA; 2 Department of Medicine, University of California San Diego, La Jolla, CA 92093, USAj-fujiki@rakuno.ac.jp (J.F.);; 3 Department of Veterinary Medicine, Rakuno Gakuen University, Ebetsu 069-8501, Hokkaido, Japan; 4 Department of Gastroenterology and Hepatology, University Hospital Cologne, 50937 Cologne, Germany; 5 Faculty of Medicine, University of Cologne, 50931 Cologne, Germany; 6 Department of Hepatology and Gastroenterology, Campus Virchow Clinic and Campus Charité Mitte, Charité University Medicine, 13353 Berlin, Germany; 7 Department of Medicine, VA San Diego Healthcare System, San Diego, CA 92161, USA; 8 Division of Gastroenterology, Hepatology & Nutrition, Rady Children’s Hospital San Diego, San Diego, CA 92123, USA

**Keywords:** butyrate, gut microbiota, MASLD, SCFAs

## Abstract

The gut microbiota plays an important role in the pathogenesis of metabolic dysfunction-associated steatotic liver disease (MASLD). In this study, we aimed to evaluate the role of the butyrate-producing bacterium *Faecalibacterium prausnitzii* in MASLD and whether supplementation with butyrate-producing bacteria, in particular *Faecalibacterium prausnitzii*, can ameliorate diet-induced steatohepatitis in mice. The relative abundance of the genus *Faecalibacterium* and its most abundant strain *Faecalibacterium prausnitzii* was determined by 16S rRNA sequencing and quantitative polymerase chain reaction (qPCR), respectively, in 95 participants with MASLD and 19 healthy control subjects. Butyrate and butyrate-producing bacteria (*Faecalibacterium prausnitzii* and *Coprococcus comes*) were gavaged to C57BL/6 mice fed a steatohepatitis-inducing diet. The fecal relative abundance of *Faecalibacterium* and *Faecalibacterium prausnitzii* was decreased in subjects with MASLD versus healthy controls and lower in individuals with MASLD and stage 3–4 fibrosis versus those with stage 0–2 fibrosis. Sodium-butyrate supplementation improved hepatic steatosis in mice on high-fat diet (HFD). Gavage of various butyrate-producing bacteria including *Faecalibacterium prausnitzii* and *Coprococcus comes* isolated from humans did not improve HFD-induced liver disease in mice. Although the abundance of *Faecalibacterium prausnitzii* is associated with MASLD severity in humans, its gavage to mice does not improve experimental diet-induced liver disease.

## 1. Introduction

Metabolic dysfunction-associated steatotic liver disease (MASLD) has emerged as a major global health concern, with its prevalence rising significantly over recent decades, now affecting 5–10% of the pediatric population and up to 38% of the adult population globally [1,2]. Therapeutic strategies for managing MASLD and its more severe form, metabolic dysfunction-associated steatohepatitis (MASH), involve a combination of lifestyle changes and pharmacological interventions aimed at weight loss [3,4,5,6,7]. Recently, the selective thyroid hormone receptor beta agonist resmetirom has shown promise in treating MASH, with positive effects on liver fat reduction and fibrosis improvement, leading to FDA approval as the first medication for certain stages of liver disease in 2024 [8,9]. Despite this advancement, effective pharmacological treatment options for the full range of MASLD are still lacking.

Short-chain fatty acids (SCFAs) are a group of organic compounds composed of a carboxyl group (COOH) linked to a short carbon chain ranging from one to five atoms. The primary SCFAs include formate (C1), acetate (C2), propionate (C3), butyrate (C4), and valerate (C5) [10,11]. Among them, acetate (C_2_H_4_O_2_), propionate (C_3_H_6_O_2_), and butyrate (C_4_H₈O_2_) are the most dominant, collectively accounting for about 95% of SCFAs present in the human body [12,13]. Butyrate, and SCFAs in general, are known for their wide range of health benefits across various diseases, including gastrointestinal disorders, metabolic diseases, such as diabetes, obesity, or MASLD, and neurological conditions [14,15]. SCFAs exert their effects by influencing gene expression and cellular processes through various mechanisms, including the activation of G-protein-coupled receptors (GPCRs) and the inhibition of histone deacetylases [16,17,18]. Butyrate, one of the most abundant SCFAs, is predominantly produced by gut bacteria during the fermentation of dietary fiber and resistant starches through several pathways, with the acetyl-CoA pathway serving as the main route for butyrate production [19,20]. This occurs particularly in anaerobic bacteria within the Firmicutes phylum, such as *Faecalibacterium* and *Coprococcus* species [21,22,23].

Given the increasing prevalence of MASLD and its limited treatment options, new therapeutic strategies for this disease must be explored. With the raising interest in the gut–liver axis and the impact of the gut microbiota on liver disease, the aim of this study was to evaluate the effects of butyrate and butyrate-producing bacteria in a mouse model of high-fat diet (HFD)-induced steatohepatitis.

## 2. Materials and Methods

### 2.1. Graphical Abstract

The upper illustrations of the graphical abstract were created with a license from https://BioRender.com.

### 2.2. Study Subjects

The study cohort was described in detail previously [24,25,26] and is shown in Table S1. In brief, MASLD was diagnosed based on the following criteria: (i) evidence of hepatic steatosis detected through liver imaging and/or histological analysis of a liver biopsy showing more than 5% of hepatocytes containing fat, (ii) daily alcohol intake below 10 g for females and 20 g for males, (iii) absence of regular use of medications known to induce fatty liver, (iv) exclusion of other conditions leading to secondary steatosis, (v) absence of other chronic liver diseases, and presence of at least one cardiometabolic risk factor, as defined by Rinella et al. [27]. Inclusion criteria for the healthy controls were (i) no history of any chronic disease, (ii) body mass index (BMI) < 25 kg/m^2^, (iii) daily alcohol intake below 10 g in females and below 20 g in males, (iv) abdominal ultrasound without abnormalities, and (v) all measured laboratory parameters within the reference ranges. Metabolic syndrome was defined according to the International Diabetes Foundation criteria [28]. Type 2 diabetes was defined as glycated hemoglobin (HbA1c) ≥ 48 mmol/mol Hb and/or fasting glucose ≥ 126 mg/dL and/or use of antidiabetic medications. Overweight was classified as BMI ≥ 25 kg/m^2^. Arterial hypertension was diagnosed based on an office blood pressure of ≥140/90 mmHg in at least two measurements on separate occasions or antihypertensive drug treatment. Blood samples for laboratory analyses were collected in the fasting state and processed following standard laboratory procedures. Liver biopsies were conducted and assessed as previously described in detail [24,25,26]. Stool donors for isolated bacterial strains are listed in Table S2. The study protocols received approval from the local ethics committee, and all participants provided written informed consent.

### 2.3. Faecalibacterium Abundance by 16S rRNA Sequencing

Stool processing and 16S rRNA sequencing were conducted as previously described [24]. In brief, DNA was extracted from frozen stool samples using the RNeasy Power Microbiome Kit (Qiagen, Hilden, Germany). Seven of the nine variable regions of the bacterial 16S rRNA gene (pool 1: V2, V4, and V8; and pool 2: V3, V6/7, and V9) were amplified using the Ion 16S Metagenomics Kit (Thermo Fisher Scientific, Waltham, MA, USA) with two primer pools (An integrated research solution for bacterial identification using 16S rRNA gene sequencing on the Ion PGM™ System with Ion Reporter™ software (version 5.10.5.0); https://www.thermofisher.com/content/dam/LifeTech/Documents/PDFs/Ion-16S-Metagenomics-Kit-Software-Application-Note.pdf (accessed 14 August 2024)). Amplicons were pooled and purified using the NucleoMag NGS Clean-up (Macherey-Nagel, Düren, Germany). Metagenomic sequencing was performed on the Ion Torrent S5 system (Thermo Fisher Scientific, Waltham, MA, USA), which operates based on pH detection. Single-end Ion Torrent sequencing reads were analyzed using the proprietary Ion Reporter (version 5.10.5.0) platform with the Metagenomics 16S w1.1 workflow. Briefly, unique sequences of at least 150 nucleotides length, and at least 10 read counts were aligned to the composite Curated MicroSEQ^®^ 16S Reference Library (version 2013.1) and Curated GreenGenes (version 13.5) database requiring 90% sequence coverage; taxonomy assignment required 97% sequence identity for genus level. The sequencing data have been deposited in the National Center for Biotechnology Information (NCBI) Sequence Read Archive under BioProject PRJNA540738, as described previously [24]. The raw data were processed using R (version 3.5.1) with the phyloseq package (version 1.28.0). The feature table in biom format was imported using the function import_biom, and the resulting phyloseq-object was merged with patient (sample) data using the merge_phyloseq function. Alpha diversity was assessed using the estimate_richness function, which utilizes the functions estimateR and diversity from the vegan package (version 2.5-6) to calculate observed operational taxonomic units and Shannon Index, respectively.

### 2.4. Mice

Male C57BL/6 mice were bred at Charles River Laboratories (Wilmington, MA, USA) and transferred to the University of California San Diego (UCSD) at 6–11 weeks of age. Mice were fed a high-fat diet (HFD) (S3282; containing 36.0% kcal from fat, 35.7% kcal from carbohydrate, 20.5% from protein, 5.49 kcal/g; Bio-Serv Ingredients, Flemington, NJ, USA), a Western diet (WD) (TD.200289; containing 41.9% kcal from fat, 43.0% kcal from carbohydrate, 15.2% from protein, 4.6 kcal/g; Teklad Diets, Madison, WI, USA), or a choline-deficient L-amino acid-defined high-fat diet (CDAA-HFD) (A06071302; containing 61% kcal from fat, 21% kcal from carbohydrate, 18% from protein, 5.2 kcal/g; 0.1% methionine and no added choline; Research Diets, New Brunswick, NJ, USA) for 4 weeks. As a control, mice were fed an irradiated low-fat control diet (“chow diet”) (TD.110637; containing 13.0% kcal from fat, 67.9% kcal from carbohydrate, 19.1% kcal from protein, 3.6 kcal/g; Teklad Diets, Madison, WI, USA) for 4 weeks. The diet was replenished weekly, and daily food intake was quantified by dividing the total amount consumed over one week by seven and normalizing it to the mice’s body weight at the time of replenishment. We documented the body weight of the mice every week. For the HFD feeding model with sodium-butyrate (Na-butyrate), mice were gavaged with 10 μL/g body weight (BW) of Na-Butyrate (400 mg/kg) or phosphate-buffered saline (PBS) 5 times per week, starting at day 1. For the CDAA-HFD and WD feeding model with Na-butyrate, mice were gavaged with 10 μL/g BW of Na-Butyrate (200 mg/kg for week 1–2; 400 mg/kg for week 3–4) or PBS 3 times per week (for week 1–2) or 5 times per week (for week 3–4), starting at day 1. For the HFD feeding models with bacteria, mice were gavaged with 200 μL of *Faecalibacterium prausnitzii* strains FP1, FP2, or *Coprococcus comes* (CC) (10^4^–10^6^ CFUs) or PBS 5 times per week, starting at day 1. Two to three biological replicates were used in each mouse feeding experiment. At the start of the study, mice were randomly assigned to different groups. They were housed in Sentry SPP systems (Allentown, NJ, USA) under a 12 h light/12 h dark cycle, with all procedures conducted during the light phase.

### 2.5. Bacteria

Bacterial strains were isolated from human stool samples under anaerobic conditions (dual gas system: nitrogen (N_2_) and anaerobic gas mixture (ANO2) (80% N_2_, 10%H_2_, 10% CO_2_ gas)) using the Whitley A25 Anaerobic Workstation (Don Whitley Scientific, Bingley, West Yorkshire, UK). Bacteria were grown in LYBHI culture medium (Brain–heart infusion medium supplemented with 0.5% yeast extract) as described by Lenoir et al. and plated on M2GSC agar plates (modified Med2 of Hobson, containing glucose, starch and cellobiose) as per Miyazaki et al. [29,30]. All substances were pre-incubated in the anaerobic chamber for 24 h at 37 °C before use. Bacterial growth was assessed by measuring the OD600 from each individual sample.

### 2.6. Real-Time Quantitative PCR

RNA was extracted from liver tissue using Trizol (Invitrogen, Carlsbad, CA, USA) and treated with DNase using the DNA-free DNA removal kit (Ambion, Austin, TX, USA). Complementary DNA (cDNA) was synthesized using the high-capacity cDNA revere transcription kit (Applied Biosystems, Foster City, CA, USA) [31]. Primer sequences for mouse genes (18S, interleukin 1ß (*IL-1ß*), chemokine (C-X-C motif) ligand 2 (*Cxcl-2*)) and bacterial genes (16S, *F. prausnitzii*) are listed in Table S3. Gene expression was analyzed by quantitative PCR using Sybr Green (Bio-Rad Laboratories, Hercules, CA, USA) on an ABI StepOnePlus real-time PCR system (Applied Biosystems, Foster City, CA, USA) and normalized relative to 18S RNA levels. To quantify the bacterial load of *Faecalibacterium prausnitzii* present in the feces, the qPCR value for each sample was multiplied by the total DNA content per gram of feces, as previously described [32].

### 2.7. Histological Staining Procedures

Histological procedures were carried out as previously described [33]. In brief, formalin-fixed tissue samples were embedded in paraffin (Paraplast plus, McCornick Scientific, Berkley, MO, USA), 5 μm sections were cut and stained with Hematoxylin and Eosin (Surgipath Medical Industries, Richmond, IL, USA). Lipid accumulation was assessed by embedding liver sections in OCT compound, cutting 10 μm frozen sections, and staining them with Oil Red O (Sigma-Aldrich, St. Luis and Burlington, MA, USA).

### 2.8. Biochemical Analysis

Hepatic triglyceride levels were quantified with the Triglyceride Liquid Reagents Kit (Pointe Scientific, Canton, MI, USA) following the company’s user manual. Plasma ALT levels were quantitated following a protocol described previously [34]. Butyrate concentrations were measured using the General Butyric Acid ELISA Kit (ABclonal Technology, Woburn, MA, USA) following the company’s user manual. For the fecal butyrate concentration measurement, the fecal samples were weighed, diluted 50× with sterile PBS and thoroughly mixed for 30 s using a bead-beater. For quantification by ELISA, 50 µL of diluted samples and 50 µL of diluted biotinylated antibody were added to wells for incubation for 1 h at 37 °C. After 5× washing, 50 µL of avidin-HRP solution was added to each well and incubated at 37 °C for 1h followed by 5× washing. Next, 50 µL of substrate solution A and 50 µL of substrate solution B were added and incubated for 10 min at 37 °C. After adding 50 µL stop solution, the optical density was measured at 450 nm.

### 2.9. Random Amplified Polymorphic DNA

RAPD fingerprinting was performed similarly to methods described before by Torriani et al. and Akopyanz et al. [35,36]. In brief, a PCR was performed using oligonucleotide primer 1254 (CCGCAGCCAA) with the following program: initiation at 94 °C for 2 min, followed by 40 cycles consisting of 94 °C for 1 min, 45 °C for 20 s, and 72 °C for 2 min and final extension at 72 °C for 5 min.

### 2.10. Whole Genome Sequencing and Sanger Sequencing

Whole genome long reads sequencing using Oxford Nanopore Technologies sequencers as well as 16S Sanger sequencing was performed by Eurofins Genomics (Lancaster, PA). DNA was isolated using the QIAamp Fast DNA Stool Mini Kit and QIAquick PCR Purification Kit per the manufacturer’s instructions (Qiagen, Hilden, Germany).

### 2.11. Statistical Analysis

Results are expressed as mean ± s.e.m. Significance was evaluated using One-way analysis of variance (ANOVA) with Holm–Šídák’s post hoc correction for more than two groups, or unpaired student *t*-test for two groups, if the values passed the Kolmogorov–Smirnov normality test. If the Kolmogorov–Smirnov normality test could not be calculated due to small n or if it failed, the Kruskal–Wallis test with Dunn’s post hoc correction or Mann–Whitney test, respectively, was employed to determine significance, unless denoted differently in the figure legends, as described before [37]. A *p* value (or adjusted *p* value after post hoc correction for more than two groups) < 0.05 was considered to be statistically significant. Statistical analyses were performed using R statistical software, R version 4.4.2 and RStudio version 2024.12.0 + 467 for Mac, 2020 the R Foundation for Statistical Computing, and GraphPad Prism 8.4.0 for Mac.

## 3. Results

### 3.1. Demographics and Clinical Data

A total number of 114 individuals, comprising 46.5% male and 53.5% female participants, were included in the study population. Of these, 95 had metabolic dysfunction-associated steatotic liver disease (MASLD), and 19 individuals without obesity or another known disease served as controls. The median age was 31.6 years in the control cohort and 53.9 years in the MASLD cohort. The median body mass index (BMI) was recorded at 20.7 kg/m^2^ in the control group and 30.2 kg/m^2^ in the MASLD group, while the median waist circumference measured 83.0 cm and 108.0 cm, respectively. Key comorbidities in the MASLD cohort included overweight (87.2%), diabetes (23.2%), arterial hypertension (64.2%), and metabolic syndrome (42.2%). Moreover, liver disease markers were measured in the plasma. Specifically, the median alanine aminotransferase (ALT) was 44.0 U/L in the MASLD group, compared to 13.5 U/L in controls. Similarly, aspartate aminotransferase (AST) levels were elevated in the MASLD group (35.0 U/L) compared to the control group (24.0 U/L). ɣ-Glutamyltransferase (GGT) levels also showed a notable increase, with a median of 72.0 U/L in the MASLD group versus 15.5 U/L in the control group. Lastly, alkaline phosphatase (AP) was higher in the MASLD group (73.0 U/L) compared to 55.0 U/L in the control group**.** Further, FibroScan results indicated a median controlled attenuation parameter (CAP) of 195 dB/m in the control group and 288 dB/m in the MASLD group and median liver stiffness of 4.45 kPa and 6.10 kPa, respectively. The median NAFLD activity score (NAS), a histological scoring system used to assess the severity of MASLD, was 4 in the subjects with MASLD (Table S1).

### 3.2. The Fecal Relative Abundance of Faecalibacterium Is Decreased in Patients with MASLD and in Particular with Advanced Fibrosis

**Figure 1:**
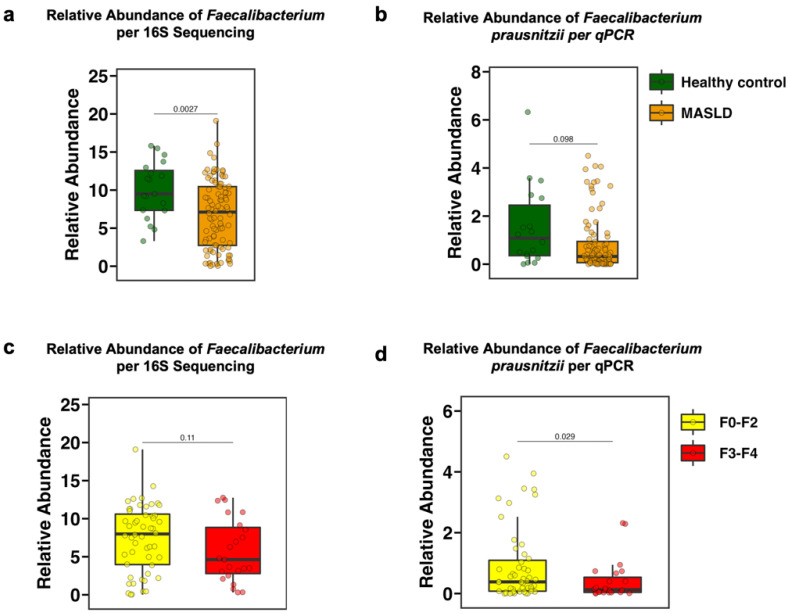
Correlation between MASLD with or without fibrosis and the relative abundance of *Faecalibacterium*. Relative abundance of *Faecalibacterium* was measured in stool samples from 114 individuals (19 controls and 95 patients with MASLD). (**a**,**b**) Relative abundance of *Faecalibacterium* (**a**) and *Faecalibacterium prausnitzii* (**b**) in 18–19 controls and 93–94 patients with MASLD. (**c**,**d**) Relative abundance of *Faecalibacterium* (**c**) and *Faecalibacterium prausnitzii* (**d**) in 51–52 patients with MASLD and fibrosis stage F0–F2, and 23 patients with MASLD and fibrosis stage F3–F4. In the box and whisker plot (**a**–**d**), the box represents the interquartile range (IQR) from the 25th to the 75th percentile, with the center line denoting the median; the lower whiskers extend to the minimum values and the top whiskers represent the 75th percentile plus 1.5-fold the interquartile distance (the distance between the 25th and 75th percentiles). *p* values were determined by student *t*-test. MASLD, metabolic dysfunction-associated steatotic liver disease; qPCR, quantative polymerase chain reaction.

We assessed the relative abundance of *Faecalibacterium* in the feces by two different methodologies to confirm the results. The relative abundance of the genus *Faecalibacterium,* as measured by 16S rRNA sequencing in stool samples from the study population, was significantly lower in patients with MASLD compared with healthy controls (*p* = 0.0027) (Figure 1a). These results were confirmed by 16S real-time quantitative polymerase chain reaction (qPCR) targeting *Faecalibacterium prausnitzii* (*F. prausnitzii*), the best-known species within the *Faecalibacterium* genus and one of the most abundant bacterial species in the human gut [38] (not significant, *p* = 0.098) (Figure 1b). Furthermore, the relative abundance of *Faecalibacterium* was non-significantly decreased in patients with advanced fibrosis (F3–F4) compared with those with no or mild fibrosis (F0–F2) (*p* = 0.11) (Figure 1c). The relative abundance of *F. prausnitzii* per qPCR was significantly decreased (*p* = 0.029) (Figure 1d). Additionally, the abundance of *Faecalibacterium* correlated with histological liver disease markers. Individuals with the top 50% of *Faecalibacterium* abundance had significantly lower NAS scores compared with those with the bottom 50% abundance (Figure S1a). Moreover, all three key components of the NAS—hepatic steatosis, lobular inflammation, and hepatic ballooning—were decreased in individuals with the top 50% of *Faecalibacterium* abundance (Figure S1b–d). Liver disease markers measured in plasma samples, including AST (*p* = 0.048), ALT (*p* = 0.11), GGT (*p* = 0.057), and AP (*p* = 0.55), were also negatively correlated with the relative abundance of *F. prausnitzii* (Figure S1e–h).

### 3.3. Sodium-Butyrate Reduces HFD-Induced Steatohepatitis

**Figure 2:**
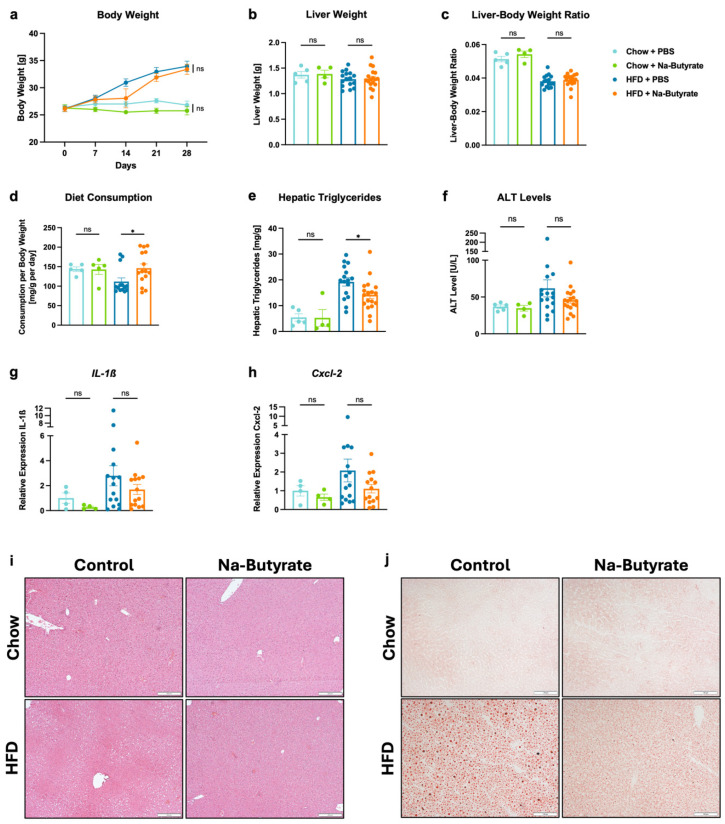
Effects of sodium-butyrate on HFD-induced steatohepatitis in mice. C57BL/6 mice were placed on a chow diet (*n* = 4–5) or HFD (*n* = 16–18) and gavaged with sodium-butyrate or PBS as a control for 4 weeks. (**a**) Absolute body weight. (**b**) Liver weight. (**c**) Liver weight-to-body weight ratio. (**d**) Daily food consumption in mg per g body weight per day. (**e**) Hepatic triglycerides. (**f**) Plasma ALT levels. (**g**,**h**) Hepatic mRNA expression of (**g**) *IL-1ß*, and (**h**) *Cxcl-2*. (**i**,**j**) Representative liver sections after (**i**) Hematoxylin and Eosin staining (bar size = 200 μm) and (**j**) after Oil Red O staining (bar size = 100 μm). Results are shown as mean ± s.e.m. *p* values were calculated using One-way ANOVA with Holm’s post hoc test (**a**–**h**). * *p* < 0.05. ns indicates *p* > 0.05. ALT, alanine aminotransferase; Cxcl-2, chemokine (C-X-C motif) ligand 2; HFD, high-fat diet; Il-1ß, interleukin 1ß; *PBS*, phosphate-buffered saline.

C57BL/6 wild-type mice were subjected to feeding of a high-fat diet (HFD), Western diet (WD), or choline-deficient L-amino acid-defined high-fat diet (CDAA-HFD) over 4 weeks. After 4 weeks of HFD, the body weight was approximately 28.43% higher compared to mice on a chow diet (Figure 2a), while the liver weight was about 6.25% lower (Figure 2b). Consequently, the liver–body weight ratio was 0.038 in the HFD group and 0.052 in the chow group (Figure 2c). Orogastric gavage with sodium-butyrate (Na-butyrate) did not lead to significant changes in body weight (*p* = 0.863), liver weight (*p* = 0.979), or liver–body weight ratio (*p* = 0.508) compared with phosphate-buffered saline (PBS) gavage in HFD-fed mice (Figure 2a–c). Mice on the chow diet consumed 143.59 mg/g BW per day in the PBS-treated group and 142.81 mg/g BW per day in the Na-butyrate-treated group. In HFD-fed mice treated with PBS, food intake was 111.83 mg/g BW per day, while food intake was significantly higher in Na-butyrate-treated HFD mice, at 146.15 mg/g BW per day (Figure 2d). Despite the increased food intake of HFD, hepatic triglycerides were significantly lower (*p* = 0.039) in Na-butyrate-treated mice versus their PBS-treated counterparts (14.19 mg/g vs. 19.23 mg/g) (Figure 2e). Additionally, plasma alanine aminotransferase (ALT) levels were non-significantly (*p* = 0.263) reduced in the Na-butyrate HFD-fed group (45.69 U/L) compared with the control HFD-fed group (61.72 U/L) (Figure 2f). Furthermore, Na-butyrate decreased hepatic inflammation in HFD-fed mice, as evidenced by lower hepatic mRNA expression of interleukin-1ß (IL-1ß) (*p* = 0.358) and chemokine (C-X-C motif) ligand 2 (CXCL2) (*p* = 0.222) compared with HFD-fed controls, although the difference was not statistically significant (Figure 2g,h). Reduced liver fat content in the HFD-fed Na-butyrate group relative to their HFD-fed controls was confirmed by Hematoxylin and Eosin staining (H&E staining) as well as Oil Red O staining of liver sections (Figure 2i,j). In the WD-based mouse model, on the other hand, we did not observe notable differences in liver disease markers, including liver–body weight ratio (*p* = 0.779), hepatic triglycerides (*p* = 0.492), and ALT levels (*p* = 0.613), or in the histological analysis between the Na-butyrate group and the control group (Figure S2a–h). In the CDAA-HFD model, treatment with Na-butyrate did not result in marked differences in other liver disease markers when compared with the control group either (Figure S3a–h). However, the liver–body weight ratio was significantly lower (*p* = 0.018) in mice treated with Na-butyrate in comparison with their CDAA-HFD-fed counterparts (Figure S3c). Based on these results, the HFD model seemed most sensitive to butyrate supplementation and was selected for further experiments.

### 3.4. Limited Effects of Orogastric Gavage with Faecalibacterium prausnitzii on Liver Function and Metabolism in HFD-Fed Mice

**Figure 3:**
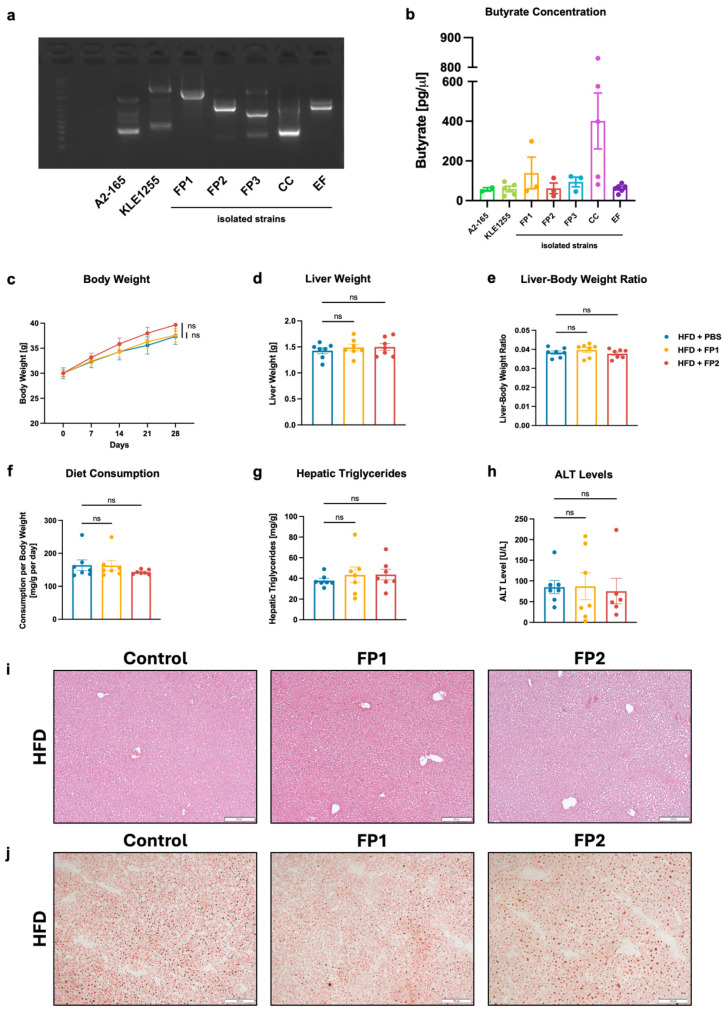
Isolation and characterization of butyrate-producing bacteria from human stool samples and impact of *Faecalibacterium prausnitzii* gavage on HFD-induced steatohepatitis in mice. C57BL/6 mice were placed on a HFD (*n* = 16–18) and gavaged with 10,000 CFUs/gavage of isolated *F. prausnitzii* strains (FP1 or FP2) or PBS as a control for 4 weeks. (**a**) RAPD fingerprinting. (**b**) Butyrate ELISA. (**c**) Absolute body weight. (**d**) Liver weight. (**e**) Liver weight-to-body weight ratio. (**f**) Daily food consumption in mg per g body weight per day. (**g**) Hepatic triglycerides. (**h**) Plasma ALT levels. (**i**,**j**) Representative liver sections after (**i**) Hematoxylin and Eosin staining (bar size = 200 μm) and (**j**) after Oil Red O staining (bar size = 100 μm). Results are shown as mean ± s.e.m. *p* values were calculated using One-way ANOVA with Holm’s post hoc test (**c**–**h**). ns indicates *p* > 0.05. ALT, alanine aminotransferase; CC, isolated *Coprococcus comes* strain; EF, isolated *Enterococcus faecalis* strain; FP1/2/3, isolated *Faecalibacterium prausnitzii* strains; HFD, high-fat diet; PBS, phosphate-buffered saline.

In the next step, butyrate-producing bacteria were isolated from human stool samples obtained from four individuals in an anaerobic chamber. Bacterial strains were selected based on the appearance of their colony-forming units (CFUs) on M2GSC agar plates (Figure S4) and identified by 16S Sanger sequencing. Additionally, random amplified polymorphic DNA (RAPD) [35,36], a type of polymerase chain reaction (PCR), was used to create a fingerprint of bacterial DNA confirming the diversity between different strains (Figure 3a). Two *Faecalibacterium prausnitzii* laboratory strains (A2-165 and KLE1255), used as references, and five isolated bacterial strains collected from four different individuals were selected for further experiments (Table S2). These included three *Faecalibacterium prausnitzii* strains (FP1, FP2, and FP3), one *Coprococcus comes* strain (CC), and one *Enterococcus faecalis* strain (EF). The identity of strain CC was confirmed by whole genome sequencing [39] as the 16S Sanger sequencing did not provide conclusive results. The mean butyrate production of strains A2-165 and KLE1255 was 56.61 pg/µL and 58.33 pg/µL, respectively, which was similar to that of FP2 at 62.22 pg/µL and EF at 62.11 pg/µL. FP3 produced slightly more butyrate, at 93.96 pg/µL, while FP1 produced 139.12 pg/µL, approximately 2.4 times the quantity produced by the laboratory strains. CC produced the most butyrate with 401.22 pg/µL, about 7 times as high as the butyrate production of *F. prausnitzii* strains A2-165 and KLE1255 (Figure 3b). Focusing on *F. prausnitzii*, two *F. prausnitzii* strains were selected based on their butyrate concentration and hence production: FP1, a high-butyrate-producing bacterium, and FP2, a low-butyrate-producing bacterium. These strains were used to evaluate their effects on liver function and metabolism in a mouse model. Wild-type mice without antibiotic pre-treatment were used, so not to disturb the gut microbiota. After 4 weeks of HFD and treatment with PBS or a low dose (10,000 CFUs per gavage) of FP1 or FP2, respectively, we did not observe significant differences in body weight, liver weight, or liver–body weight ratio between mice that received PBS and those treated with FP1 or FP2 (Figure 3c–e). Additionally, the diet consumption per body weight was comparable across all three groups (Figure 3f). Further, we did not find significant differences in liver disease and inflammatory markers, such as hepatic triglycerides and ALT levels (Figure 3g,h). These findings were consistent with H&E and Oil Red O staining of liver sections, which showed similar amounts and sizes of fat droplets across the different groups (Figure 3i,j).

### 3.5. High-Dose Gavage of Faecalibacterium prausnitzii Shows No Significant Impact on HFD-Induced Liver Disease in Mice

**Figure 4:**
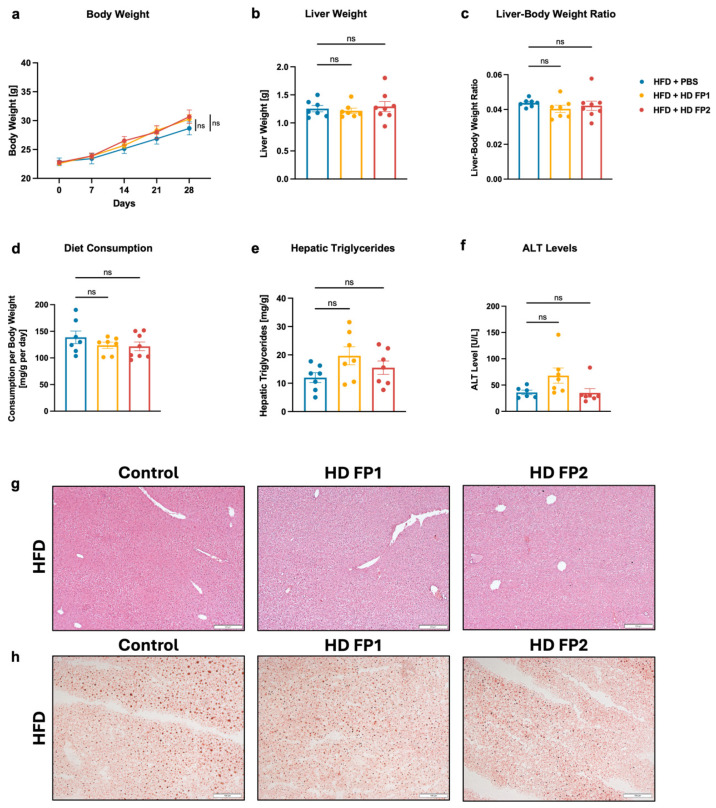
Impact of high-dose *Faecalibacterium prausnitzii* gavage on HFD-induced steatohepatitis in mice. C57BL/6 mice were placed on a HFD (*n* = 7) and gavaged with 1,000,000 CFUs/gavage of isolated *F. prausnitzii* strains (FP1 or FP2) or PBS as a control for 4 weeks. (**a**) Absolute body weight. (**b**) Liver weight. (**c**) Liver weight-to-body weight ratio. (**d**) Daily food consumption in mg per g body weight per day. (**e**) Hepatic triglycerides. (**f**) Plasma ALT levels. (**g**,**h**) Representative liver sections after (**g**) Hematoxylin and Eosin staining (bar size = 200 μm) and (**h**) after Oil Red O staining (bar size = 100 μm). Results are shown as mean ± s.e.m. *p* values were calculated using One-way ANOVA with Holm’s post hoc test (**a**–**f**). ns indicates *p* > 0.05. ALT, alanine aminotransferase; HD FP1/2, high-dose of isolated *Faecalibacterium prausnitzii* strains; HFD, high-fat diet; PBS, phosphate-buffered saline.

To rule out potential underdosing of FP as a possible cause of no significant difference in liver disease, the dose of FP1 and FP2 was increased to 1,000,000 CFUs per gavage in a subsequent mouse model. However, no significant differences in body weight, liver weight, or body–liver weight ratio were observed between the high-dose (HD) FP1 or FP2 groups and the control group, despite similar food intake across all three groups (Figure 4a–d). Although not statistically significant, hepatic triglyceride levels were higher in mice gavaged with HD FP1 and HD FP2 in relation to those that were gavaged with PBS (Figure 4e). Plasma ALT levels were measured at 35.96 U/L in the control group, 55.02 U/L in the HD FP1 group, and 37.84 U/L in the HD FP2 group (Figure 4f). These results were consistent with H&E and Oil Red O stained liver sections, which showed a similar phenotype across the different groups, with marginally more fat droplets in the HD FP1 group (Figure 4g,h). Furthermore, qPCR analysis revealed a significantly higher fecal load of *F. prausnitzii* in HFD-fed mice treated with FP in comparison with HFD-fed controls (Figure S5a). Fecal butyrate concentrations were significantly elevated in the HFD-fed PBS group compared with the chow-fed PBS group, while there was no statistically significant difference in the butyrate concentrations between HFD-fed mice treated with PBS and FP (Figure S5b).

### 3.6. Gavage of High-Butyrate-Producing Coprococcus Comes Does Not Improve Liver Disease in HFD-Fed Mice

**Figure 5:**
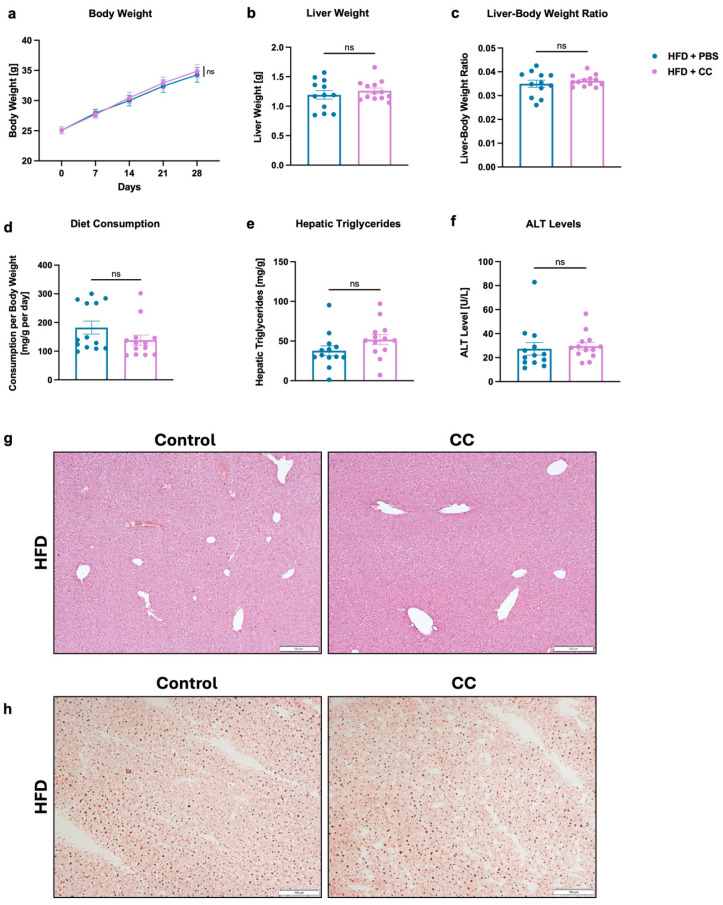
Impact of *Coprococcus comes* gavage on HFD-induced steatohepatis in mice. C57BL/6 mice were placed on a HFD (*n* = 13) and gavaged with 1,000,000 CFUs/gavage of isolated *C. comes* strain or PBS as a control for 4 weeks. (**a**) Absolute body weight. (**b**) Liver weight. (**c**) Liver weight-to-body weight ratio. (**d**) Daily food consumption in mg per g body weight per day. (**e**) Hepatic triglycerides. (**f**) Plasma ALT levels. (**g**,**h**) Representative liver sections after (**g**) Hematoxylin and Eosin staining (bar size = 200 μm) and (**h**) after Oil Red O staining (bar size = 100 μm). Results are shown as mean ± s.e.m. *p* values were calculated using Mann–Whitney test (**a**–**c**,**e**,**f**) or student *t*-test (**d**). ns indicates *p* > 0.05. ALT, alanine aminotransferase; CC, isolated *Coprococcus comes* strain; HFD, high-fat diet; PBS, phosphate-buffered saline.

In another 4-week HFD mouse feeding experiment, the effects of a clinical *Coprococcus comes* strain (CC), identified as the highest butyrate-producer among the isolated bacteria, were studied. After 4 weeks of HFD, the body weight of mice gavaged with PBS reached 34.37 g, with a liver weight of 1.19 g, while mice gavaged with CC weighed 34.94 g and had an average liver weight of 1.27 g (Figure 5a,b). Hence, no significant differences in body weight, liver weight, or liver–body weight ratio were observed between the groups (Figure 5a–c). Although food intake in the CC group was lower, hepatic triglyceride levels were non-significantly higher in these mice compared with the control group (37.40 mg/g in the PBS group vs. 51.92 mg/g in the CC group) (Figure 5d,e). Additionally, plasma ALT levels were similar in both groups (26.46 U/L in the PBS group vs. 29.20 U/L in the CC group) (Figure 5f). The histological analysis of liver tissue, performed using H&E and Oil Red O staining, was consistent with previous results and showed no differences between the groups (Figure 5g,h).

## 4. Discussion

In the present study, we demonstrate that the relative abundance of *Faecalibacterium*, one of the most abundant bacteria in the human gut [38], is decreased in patients with metabolic dysfunction-associated steatotic liver disease (MASLD) (Figure 1a,b). We further observe a decrease in *Faecalibacterium* abundance in patients with more advanced fibrosis compared to patients with less severe fibrosis (Figure 1c,d). These findings align with several previous studies regarding the relationship between *Faecalibacterium*, particularly *Faecalibacterium prausnitzii* (*F. prausnitzii*), and MASLD [40]. Thus, Yang et al. state that several bacterial genera, including *Faecalibacterium*, *Subdoligranulum*, *Haemophilus*, and *Roseburia* are decreased across different subtypes of MASLD [41]. Other studies show that both, obese and lean patients with metabolic dysfunction-associated steatohepatitis (MASH), an advanced form of MASLD, have a decreased abundance of *Faecalibacterium* [42,43,44,45]. Moreover, previous studies not only show a link between the severity of fibrosis and the gut microbiota, but also define a profile of bacterial features including a decrease in *Faecalibacterium* abundance to detect cirrhosis, the most severe and extensive form of fibrosis, in patients with MASLD [46,47,48,49]. Further, the correlation between MASLD risk indicators and the abundance of *Faecalibacterium* is consistent with previous findings. In a recent scientific report published in nature, Yang et al. report a negative correlation between magnetic resonance imaging-proton density fat fraction (MRI-PDFF), aspartate aminotransferase (AST), alanine aminotransferase (ALT), body mass index (BMI) as well as obesity class and *Faecalibacterium* [41].

However, the imbalance between the MASLD group (*n* = 95) and the healthy control group (*n* = 19) may have affected the statistical power and reliability of between-group comparisons, posing a limitation when interpreting the findings. The small number of healthy controls also limits the generalizability of the results, as they may not fully represent the broader population. Future studies with larger and more balanced sample sizes are needed to validate these findings.

In a mouse model, we further demonstrate that HFD-fed mice have lower hepatic triglycerides and liver fat content on histology, central markers of murine metabolic liver disease [50], when treated with Na-butyrate (Figure 2e,i,j). They also show a decrease in inflammatory markers in comparison with their controls (Figure 2f–h). These results are consistent with recent studies from other research groups regarding the treatment of high-fat diet (HFD)-induced steatohepatitis with Na-butyrate or tributyrin, a precursor to butyrate. Several studies reported a decrease in triglycerides and cholesterol in the liver, as well as a decrease in pro-inflammatory genes, such as *Tnf-α*, *IL-1ß*, *IL-2*, or *IL-6*, and the liver cell injury marker ALT [51,52,53]. In the present study, we do not observe a difference in body weight between mice in the Na-butyrate group and the control group (Figure 2a), though previous studies highlight that butyrate prevented obesity in HFD-fed mice [54,55,56]. Further, butyrate did not improve hepatic steatosis in mice treated with other diet, e.g., CDAA-HFD (Figure S3). One main reason may be that the other feeding models—in particular CDAA-HFD—resulted in much more liver disease than HFD, as indicated by markedly higher hepatic triglyceride levels and plasma ALT levels (Figure S3e–h), and therefore a relatively smaller improvement with butyrate supplementation might not be enough to show a significant improvement in these more severe disease models. Future studies are required to elucidate those findings.

In our study, we isolated several butyrate-producing bacterial strains, including *Faecalibacterium prausnitzii*, *Coprococcus comes* (*C. comes*) and *Enterococcus faecalis (E. faecalis)*. While *F. prausnitzii* and *C. comes* are well-known butyrate producers belonging to the Clostridium clusters IV and XIVa, respectively, *E. faecalis* is typically referred to as pathogenic [57,58]. The RAPD fingerprinting data show that each isolated bacterium belongs to a different bacterial strain (Figure 3a). This method, based on PCR and gel electrophoresis, generates a DNA fingerprint that can detect even single-base alterations. First described in 1990 by Williams et al. and Welsh et al., it is now well established not only in phylogenetic studies to distinguish between different strains, but also in genotoxicity and carcinogenesis studies [59,60,61,62,63]. Moreover, the butyrate production varies among the different strains (Figure 3b), which highlights the diversity in metabolic capabilities among butyrate-producing bacteria and is consistent with previous research articles that describe different phylogroups of *F. prausnitzii* [64]. We further show that administering isolated *F. prausnitzii* strains, which produce either high or low concentrations of butyrate, to HFD-fed mice does not result in significant changes in the phenotype of diet-induced liver disease (Figure 3c–j). Similarly, increasing the dose of those *F. prausnitzii* strains by 100 times does not lead to any improvement in the disease (Figure 4a–h), although the significantly higher fecal load of *F. prausnitzii* in HFD-fed mice treated with the bacterium compared to controls indicates a successful colonization (Figure S5a). However, fecal butyrate concentrations did not differ between mice treated with *F. prausnitzii* and their HFD-fed controls, which may be due to the fact that the intestinal microenvironment might not be conducive to increase or maintain sufficient butyrate production by *F. prausnitzii* strains in our study and may explain the lack of improvement in the disease. (Figure S5b). These results contrast with previous studies demonstrating beneficial effects of *Faecalibacterium*, in particular *F. prausnitzii*, in mouse models of MASH. Shin et al. reported that *F. prausnitzii* administration in mice on a high-fructose high-fat diet improves glucose homeostasis, prevents hepatic lipid accumulation, and reduces liver inflammation and fibrosis [65]. In another study, researchers found that oral supplementation of *F. prausnitzii* in HFD-fed mice lowers hepatic fat content, decreases liver enzymes like ALT and AST that are linked to hepatic inflammation, improves insulin sensitivity, and reduces inflammation in adipose tissue [66]. However, Hu et al. show that only 5 out of 12 orally gavaged *F. prausnitzii* strains have significant effects on liver disease in HFD-fed mice and only 2 strains are able to significantly stimulate short-chain fatty acid (SCFA) production and modulate the gut microbiota. Hu et al. also noted that the level of butyrate production and protectiveness are not strictly correlated as some lower-butyrate producers show greater protective effects than high-butyrate producers. This suggest that butyrate production is not the only factor leading to the beneficial effects of *F. prausnitzii*. Further, this study highlights the variability in the capacity of different *F. prausnitzii* strains to improve MASLD [67,68]. The diversity within the *F. prausnitzii* species observed in the study by Hu et al. is a possible explanation for the lack of improvement in the disease in our mouse model. Other potential explanations for the lack of effect in the present study include the complex interactions within the gut microbiome, as well as the complex interplay between butyrate and the human organism.

In another mouse model, we show that *C. comes* gavage does not lead to a reduction in liver disease markers, such as liver–body weight ratio, hepatic triglycerides, or ALT, even though it produces the highest levels of butyrate in relation to the bacterial strains isolated in the prior experiment and the control strains (Figure 3b and Figure 5a–f). A similar histological pattern in both the treated and the control groups further confirms the lack of disease prevention (Figure 5g,h). These findings differ from those of a recent study by Lu et al. This study from 2024 revealed a significant decrease in *Coprococcus* abundance in patients with MASLD compared to healthy controls. Furthermore, it demonstrated protective effects of *Coprococcus* in an HFD-based mouse model [69]. However, there are only few studies that investigate the effects of *Coprococcus* in relation to MASLD in diet-induced mouse models, which is why the results of our study can only be interpreted to a limited extend.

When interpreting the results, several limitations should be acknowledged. First, the relatively short duration of the mouse experiments (four weeks) may not have been sufficient to fully capture the long-term effects of butyrate or butyrate-producing bacterial supplementation on liver disease progression. Future studies with extended follow-up periods are necessary to determine whether the observed effects persist or evolve over time. Second, there was a notable inconsistency between human and animal findings. In humans, decreased *Faecalibacterium* abundance correlated with greater MASLD severity; however, supplementation with *F. prausnitzii* in mice did not lead to significant improvements. This discrepancy raises concerns about the translatability of findings between species and suggests that additional factors may influence the efficacy of bacterial supplementation in different biological contexts. Further research is needed to clarify these differences and identify conditions under which *Faecalibacterium* exerts beneficial effects. Third, the study tested only a limited number of butyrate-producing bacterial strains and doses. A broader selection of strains and doses may have produced different results, highlighting the need for future research to explore a more diverse range of butyrate-producing bacteria and their potential strain-specific effects on liver disease. Additionally, this study did not investigate the underlying mechanisms by which *Faecalibacterium* or butyrate may influence liver disease. While correlations and functional effects were observed, the specific pathways involved—such as immune modulation, metabolic regulation, or gut–liver axis interactions—remain unclear. Future studies using transcriptomic, metabolomic, and immunological approaches could help elucidate these mechanisms. Finally, there were inconsistencies in the effects of sodium-butyrate supplementation across different dietary models. While beneficial effects were observed in the high-fat diet (HFD) model, no significant improvements were seen in the western diet (WD) or choline-deficient, amino acid-defined high-fat diet (CDAA-HFD) models. The reasons for these differences remain unclear and may be related to variations in dietary composition, metabolic responses, or gut microbiome alterations. Further investigation is needed to understand these discrepancies and determine the contexts in which butyrate supplementation is most effective.

Based on these results, further research regarding the complex interactions and mechanisms within the gut microbiome and potential other factors influencing the effects of butyrate-producing bacteria on the human organism is needed. Furthermore, various mouse models, including germ-free mice, humanized mice, or conventional mice pre-treated with antibiotics, could be explored to evaluate the effects *F. prausnitzii* supplementation in mice.

## 5. Conclusions

In conclusion, our study provides important insights into the relationship between *Faecalibacterium prausnitzii* and metabolic dysfunction-associated liver disease (MASLD). We observed that the relative abundance of *Faecalibacterium* was significantly lower in individuals with MASLD compared with healthy controls, and particularly in those with advanced liver fibrosis. This finding suggests that *Faecalibacterium* could potentially serve as a biomarker for MASLD severity, although further research is needed to develop and test a system for using of *Faecalibacterium* as a molecular marker for MASLD. Further, we demonstrated the capability of butyrate to improve diet-induced steatohepatitis in a short high-fat diet (HFD)-based mouse model simulating MASLD. However, regardless of promising associations between *Faecalibacterium* levels and liver disease severity, orogastric gavage with *Faecalibacterium prausnitzii* strains did not show significant improvements in liver function or metabolic parameters in animal models, independent of the dosage. Moreover, supplementation of *Coprococcus comes* did not show positive effects on the liver in HFD-fed mice, despite its high production of butyrate. Further research into the clinical utility of the use of *Faecalibacterium prausnitzii* and other probiotics in MASLD is hence necessary.

## Data Availability

The data presented in this study are openly available in [National Center for Biotechnology Information], reference number [https://www.ncbi.nlm.nih.gov/bioproject/540738] [PRJNA540738] (accessed 14 August 2024).

## Supplementary Materials

The following supporting information can be downloaded at: https://www.mdpi.com/article/10.3390/microorganisms13030675/s1, Figure S1. Relative abundance of *Faecalibacterium prausnitzii* is correlated with liver disease markers. Histologic analysis of liver biopsies was performed and liver disease markers were quantitated in plasma samples from 114 individuals (19 controls and 95 patients with MASLD). (a–d) Individuals with the top 50% of *Faecalibacterium* abundance per 16S sequencing compared with individuals with the bottom 50% *Faecalibacterium* abundance regarding NAFLD activity score (a), steatosis grade (b), inflammation grade (c), and ballooning severity (d). (e) Pearson correlation between AST and *F. prausnitzii* abundance. (f) Pearson correlation between ALT and *F. prausnitzii* abundance. (g) Pearson correlation between GGT and *F. prausnitzii* abundance. (h) Pearson correlation between alkaline phosphatase and *F. prausnitzii* abundance. In the box and whisker plot (a–d), the box represents the interquartile range (IQR) from the 25th to the 75th percentile, with the center line denoting the median; the lower whiskers extend to the minimum values and the top whiskers represent the 75th percentile plus 1.5-fold the interquartile distance (the distance between the 25th and 75th percentiles); p values were determined by student *t*-test. Alk Phos, alkaline phosphatase; ALT, alanine aminotransferase; AST, aspartate aminotransferase; Fb, *Faecalibacterium*; *F. prausnitzii*, *Faecalibacterium prausnitzii*; GGT, ɣ-glutamyltransferase; NAFLD, non-alcoholic fatty liver disease; NAS, NAFLD Activity Score. Figure S2. Limited effects of Sodium-butyrate on liver disease in WD-fed mice. C57BL/6 mice were placed on a WD (*n* = 7–8) and gavaged with sodium-butyrate or PBS as a control for 4 weeks. (a) Absolute body weight. (b) Liver weight. (c) Liver weight-to-body weight ratio. (d) Daily food consumption in mg per g body weight per day. (e) Hepatic triglycerides. (f) Plasma ALT levels. (g,h) Representative liver sections after (g) hematoxylin and eosin staining (bar size = 200 μm) and (h) after Oil Red O staining (bar size = 100 μm). Results are shown as mean ± s.e.m. *p* values were calculated using Mann-Whitney test (a–f). ns indicates *p* > 0.05. ALT, alanine aminotransferase; WD, Western diet; PBS, phosphate-buffered saline. Figure S3. Limited effects of Sodium-butyrate on liver disease in CDAA-HFD-fed mice. C57BL/6 mice were placed on a CDAA-HFD (*n* = 7–8) gavaged with sodium-butyrate or PBS as a control for 4 weeks. (a) Absolute body weight. (b) Liver weight. (c) Liver weight-to-body weight ratio. (d) Daily food consumption in mg per g body weight per day. (e) Hepatic triglycerides. (f) Plasma ALT levels. (g,h) Representative liver sections after (g) hematoxylin and eosin staining (bar size = 200 μm) and (h) after Oil Red O staining (bar size = 100 μm). Results are shown as mean ± s.e.m. *p* values were calculated using Mann-Whitney test (a–f). * *p* < 0.05. ns indicates *p* > 0.05. ALT, alanine aminotransferase; CDAA-HFD, choline-deficient L-amino acid-defined high-fat diet; PBS, phosphate buffered saline. Figure S4. Appearance of butyrate-producing bacteria on M2GSC agar plates. 100μl of diluted (10^−3^ on the right and 10^−6^ on the left) bacterial culture media was plated on half of an M2GSC agar plate each and incubated in the anaerobic chamber for 72 h. Figure S5. Orogastric gavage of *F. prausnitzii* increases abundance of *F. prausnitzii* in mouse feces. C57BL/6 mice were fed a chow diet (*n* = 13) or HFD (*n* = 25–26) with or without an isolated *F. prausnitzii* strain for 4 weeks. (a) Burden of *Faecalibacterium prausnitzii*. (b) Butyrate concentration. Results are shown as mean ± s.e.m. p values were calculated using Kruskal-Wallis test with Dunn’s post hoc test. * *p* < 0.05. ns indicates *p* > 0.05. FP, isolated *Faecalibacterium prausnitzii* strain; HFD, high fat diet; PBS, phosphate buffered saline. Table S1. Baseline demographic and laboratory data of the study population. Values are presented as median and upper and lower quartiles in brackets. The number of subjects for which data were available is indicated in the first column. Continuous variables were compared using the Wilcoxon-Whitney-Mann rank-sum test. Categorical variables were compared using the Pearson’s Chi-squared test. Statistical significance is indicated by *p* < 0.05. AP, alkaline phosphatase; ALT, alanine aminotransferase; art., arterial; AST, aspartate aminotransferase; BMI, body mass index; CAP, controlled attenuation parameter; *F. prausnitzii*, *Faecalibacterium prausnitzii*; GGT, ɣ-glutamyltransferase; MASLD, metabolic dysfunction–associated steatotic liver disease; NAFLD, non-alcoholic fatty liver disease; NAS, NAFLD Activity Score; qPCR, real-time quantitative polymerase chain reaction. Table S2. Baseline demographic and laboratory data of the stool donors. ALT, alanine aminotransferase; AST, aspartate aminotransferase; BMI, body mass index; CC, isolated *Coprococcus comes* strain; EF, isolated *Enterococcus faecalis* strain; FP1/2/3, isolated *Faecalibacterium prausnitzii* strains; MRI-PDFF, magnetic resonance imaging-proton density fat fraction. Table S3. List of quantitative PCR primers and their sequence. Cxcl-2, chemokine (C-X-C motif) ligand 2; *F. prausnitzii*, *Faecalibacterium prausnitzii*; IL-1ß, interleukin 1ß; PCR, polymerase chain reaction. References [35,37,70,71,72,73] are cited in the supplementary materials.

## Abbreviations

ALT, alanine aminotransferase; AST, aspartate aminotransferase; AP, alkaline phosphatase; BMI, body mass index; CAP, controlled attenuation parameter; CDAA-HFD, choline-deficient L-amino acid-defined high-fat diet; CFU, colony-forming unit; CLD, chronic liver disease; GGT, ɣ-glutamyltransferase; H&E staining, Hematoxylin and Eosin staining; HD, high-dose; HFD, high-fat diet; LPS, lipopolysaccharide; MASH, metabolic dysfunction-associated steatohepatitis; MASLD, metabolic dysfunction-associated steatotic liver disease; Na-butyrate, sodium-butyrate; NAFLD, non-alcoholic fatty liver disease; NAS, NAFLD Activity Score; PBS, phosphate-buffered saline; qPCR, quantative polymerase chain reaction; RAPD, random amplified polymorphic DNA; SCFA, short-chain fatty acid; WD, Western diet.
