# Supplementary material for: Faecalibacterium prausnitzii Is Associated with Disease Severity in MASLD but Its Supplementation Does Not Improve Diet-Induced Steatohepatitis in Mice
Source: Microorganisms. 2025 Mar 18;13(3):675. doi: 10.3390/microorganisms13030675 (PMC11944644; doi:10.3390/microorganisms13030675)
Supplement: Supplementary file 1 [file microorganisms-13-00675-s001.zip › microorganisms-3480346-supplementary.pdf]

Faecalibacterium prausnitzii is associated with disease severity in MASLD but its supplementation does not improve diet-induced steatohepatitis in mice

Eliane Münte, Greta Viebahn, Amit Khurana, Jumpei Fujiki, Tomohiro Nakamura, Sonja Lang, Münevver Demir, Bernd Schnabl, Phillipp Hartmann

Table of Contents

Supplementary Tables and Figures 2
Supplementary Tables 2
Supplementary Table S1 2
Supplementary Table S2 3
Supplementary Table S3 4
Supplementary Figures 5
Supplementary Figure S1 5
Supplementary Figure S2 7
Supplementary Figure S3 9
Supplementary Figure S4 11
Supplementary Figure S5 12
Supplementary References 13

Supplementary Tables and Figures

Supplementary Tables

Table S1. Baseline demographic and laboratory data of the study population.

	N	Entire Cohort *N=114*	Healthy control *N=19*	MASLD *N=95*	P value
Age	114	52.7 [35.7;61.0]	31.6 [29.2;37.1]	53.9 [41.8;63.3]	<0.001
Gender:	114				0.867
Male		53 (46.5%)	8 (42.1%)	45 (47.4%)	
BMI [kg/m^2]	112	29.6 [25.1;32.6]	20.7 [19.5;23.5]	30.2 [27.4;33.7]	<0.001
Weight [kg]	112	83.4 [71.9;99.8]	68.0 [60.0;73.0]	87.7 [76.1;103]	<0.001
Height [cm]	112	171 [165;180]	178 [173;180]	170 [164;178]	0.002
Waist [cm]	95	104 [89.8;114]	83.0 [78.0;85.0]	108 [97.8;119]	<0.001
Overweight	112	82 (73.2%)	0 (0.00%)	82 (87.2%)	<0.001
Diabetes	113	22 (19.5%)	0 (0.00%)	22 (23.2%)	0.021
Art. Hypertension	113	61 (54.0%)	0 (0.00%)	61 (64.2%)	<0.001
Metabolic Syndrome	113	40 (35.4%)	0 (0.00%)	40 (42.1%)	0.002
ALT [U/L]	111	40.0 [25.0;66.0]	13.5 [11.2;22.0]	44.0 [33.0;76.0]	<0.001
AST [U/L]	111	32.0 [25.0;48.0]	24.0 [18.2;25.0]	35.0 [28.0;51.0]	<0.001
GGT [U/L]	111	60.0 [28.5;118]	15.5 [11.0;21.5]	72.0 [45.0;124]	<0.001
AP [U/L]	110	71.0 [59.0;92.2]	55.0 [50.0;64.0]	73.0 [63.0;94.0]	<0.001
FibroScan (CAP [dB/m])	50	267 [202;307]	195 [186;205]	288 [261;317]	<0.001
FibroScan [kPa]	108	5.45 [4.47;10.6]	4.45 [4.10;5.32]	6.10 [4.68;11.5]	0.001
NAS (Biopsy)	65	4.00 [3.00;5.00]	n/a	4.00 [3.00;5.00]	
Fibrosis Stage (Biopsy)	65	1.00 [1.00;2.00]	n/a	1.00 [1.00;2.00]	
Relative abundance of Genus *Faecalibacterium* per 16S sequencing	113	113	19 (100%)	94 (98.9%)	
Relative abundance of *F. prausnitzii* per qPCR	111	111	18 (94.7%)	93 (97.9%)	

Values are presented as median and upper and lower quartiles in brackets. The number of subjects for which data were available is indicated in the first column. Continuous variables were compared using the Wilcoxon-Whitney-Mann rank-sum test. Categorical variables were compared using the Pearson's Chi-squared test. Statistical significance is indicated by $p<0.05$. *AP*, alkaline phosphatase; *ALT*, alanine aminotransferase; *art.*, arterial; *AST*, aspartate aminotransferase; *BMI*, body mass index; *CAP*, controlled attenuation parameter; *F. prausnitzii, Faecalibacterium prausnitzii*; *GGT*, ɣ-glutamyltransferase; *MASLD*, metabolic dysfunction–associated steatotic liver disease; *NAFLD*, non-alcoholic fatty liver disease; *NAS*, NAFLD Activity Score; *qPCR*, real-time quantitative polymerase chain reaction.

Table S2. Baseline demographic and laboratory data of the stool donors.

	**Donor 1**	**Donor 2**	**Donor 3**	**Donor 4**
Gender	Female	Male	Male	Male
Age [years]	21	15	10	12
BMI [kg/m^2]	21.3	33.5	16	32.3
BMI percentile	-	98.5	31.2	99.1
Weight [kg]	65.3	96.9	34.3	89.3
Height [cm]	175.2	170.2	146.5	166.3
Other features	-	-	-	ALT 50, AST 27; hepatic fat fraction 10% per MRI-PDFF consistent with hepatic steatosis
Isolated strains	FP2, EF	FP1	CC	FP3

ALT, alanine aminotransferase; *AST*, aspartate aminotransferase; *BMI*, body mass index; *CC*, isolated *Coprococcus comes* strain; *EF*, isolated *Enterococcus faecalis* strain; *FP1/2/3*, isolated *Faecalibacterium prausnitzii* strains; *MRI-PDFF,* magnetic resonance imaging-proton density fat fraction.

Table S3. List of quantitative PCR primers and their sequence.

Name	**Forward**	**Reverse**	**Reference**
1254	CCGCAGCCAA	-	Torriani et al.[1]
16S	GTGSTGCAYGGYTGTCGTCA	ACGTCRTCCMCACCTTCCTC	Maeda et al.[2]
18S	AGTCCCTGCCCTTTGTACACA	CGATCCGAGGGCCTCACTA	Hartmann et al.[3]
Cxcl-2	AAAGTTTGCCTTGACCCTGAA	CTCAGACAGCGAGGCACATC	Roh et al.[4]
IL-1ß	GGTCAAGGTTTGGAAGCAG	TGTGAAATGCCACCTTTTGA	Niu et al.[5]
FPR-2F, Fprau645R	GGAGGAAGAAGGTCTTCGG	AATTCCGCCTACCTCTGCACT	Fitzgerald et al.[6]

Cxcl-2, chemokine (C-X-C motif) ligand 2; *F. prausnitzii, Faecalibacterium prausnitzii*; *IL-1ß*, interleukin 1ß; *PCR*, polymerase chain reaction

Supplementary Figures

Figure S1

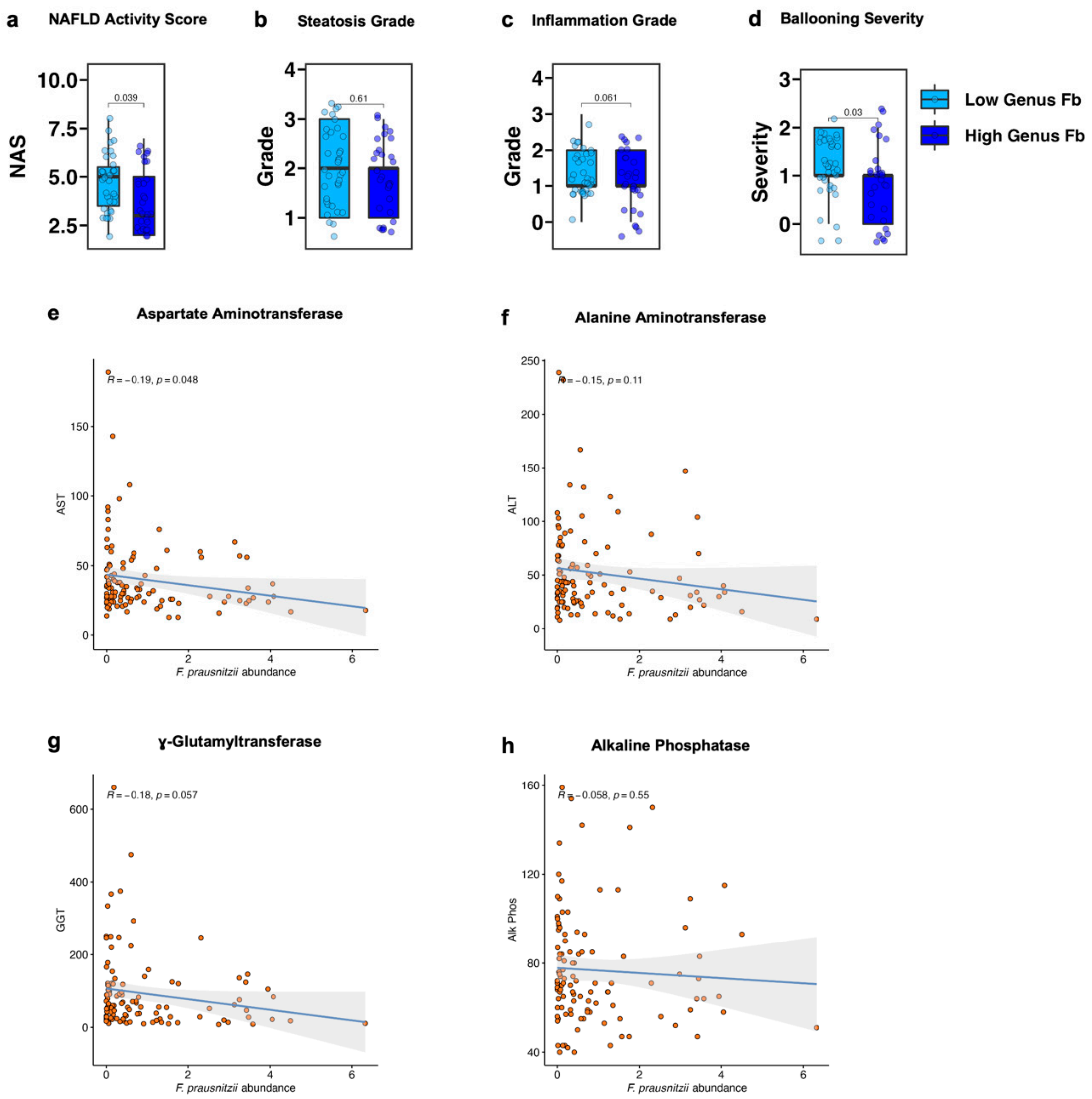

Figure S1. Relative abundance of *Faecalibacterium prausnitzii* is correlated with liver disease markers. Histologic analysis of liver biopsies was performed and liver disease markers were quantitated in plasma samples from 114 individuals (19 controls and 95 patients with MASLD). **(a-d)** Individuals with the top 50% of *Faecalibacterium* abundance per 16S sequencing compared with individuals with the bottom 50% *Faecalibacterium* abundance regarding NAFLD activity score **(a)**, steatosis grade **(b)**,

inflammation grade **(c)**, and ballooning severity **(d)**. **(e)** Pearson correlation between AST and *F. prausnitzii* abundance. **(f)** Pearson correlation between ALT and *F. prausnitzii* abundance. **(g)** Pearson correlation between GGT and *F. prausnitzii* abundance. **(h)** Pearson correlation between alkaline phosphatase and *F. prausnitzii* abundance. In the box and whisker plot **(a-d)**, the box represents the interquartile range (IQR) from the 25th to the 75th percentile, with the center line denoting the median; the lower whiskers extend to the minimum values and the top whiskers represent the 75th percentile plus 1.5-fold the interquartile distance (the distance between the 25th and 75th percentiles); p values were determined by student t-test. *Alk Phos*, alkaline phosphatase; *ALT*, alanine aminotransferase; *AST*, aspartate aminotransferase; *Fb, Faecalibacterium; F. prausnitzii, Faecalibacterium prausnitzii*; *GGT*, ɣ-glutamyltransferase; *NAFLD*, non-alcoholic fatty liver disease; *NAS*, NAFLD Activity Score.

Figure S2

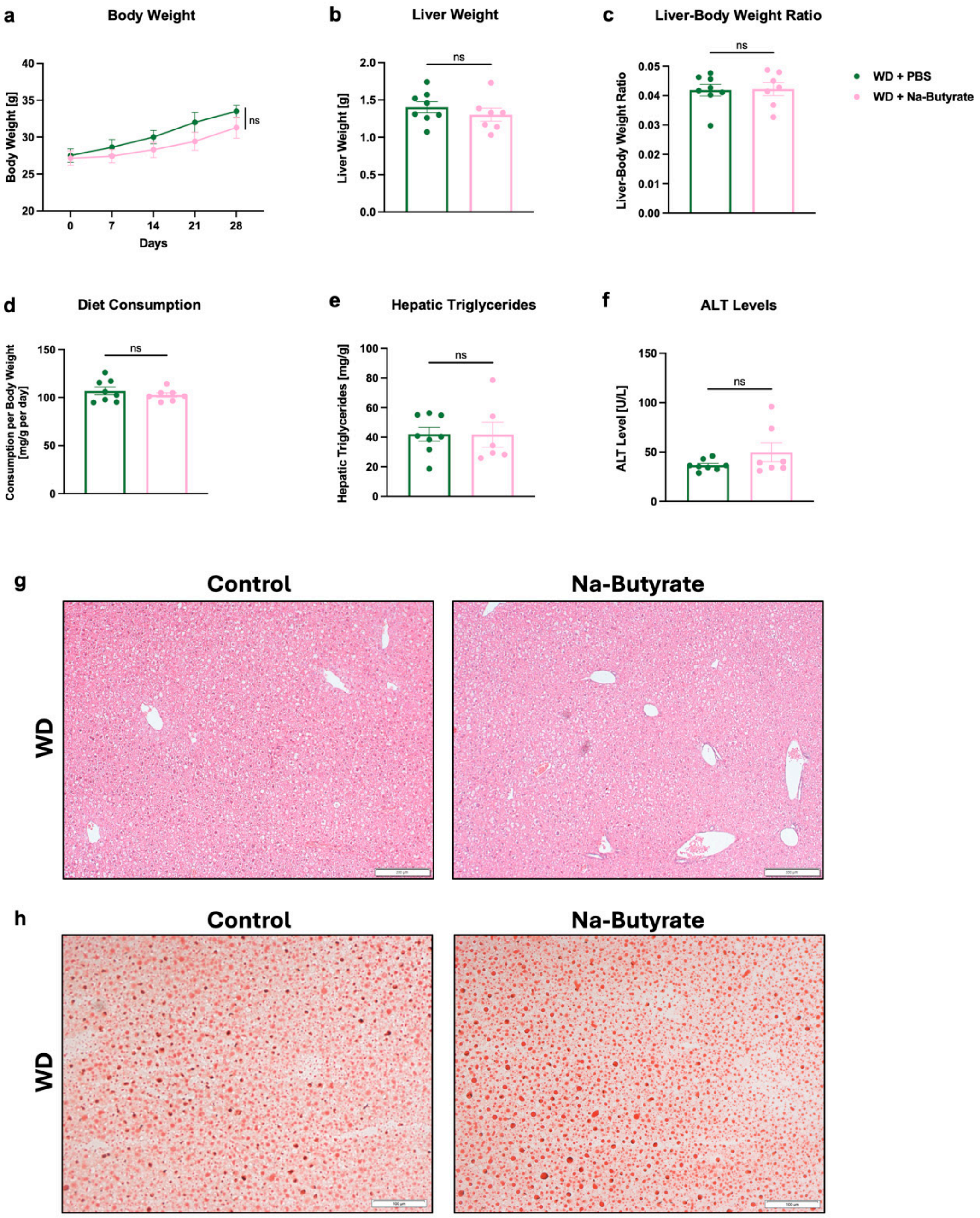

Figure S2. Limited effects of Sodium-butyrate on liver disease in WD-fed mice. C57BL/6 mice were placed on a WD (*n*=7-8) and gavaged with sodium-butyrate or PBS as a control for 4 weeks. **(a)** Absolute body weight. **(b)** Liver weight. **(c)** Liver weight-to-body weight ratio. **(d)** Daily food consumption in mg per g body weight per day. **(e)** Hepatic

triglycerides. **(f)** Plasma ALT levels. **(g–h)** Representative liver sections after **(g)** hematoxylin and eosin staining (bar size=200 μm) and **(h)** after Oil Red O staining (bar size = 100 μm). Results are shown as mean ± s.e.m. p values were calculated using Mann-Whitney test **(a–f).** ns indicates $p > 0.05$. *ALT,* alanine aminotransferase; *WD*, Western diet; *PBS*, phosphate-buffered saline.

Figure S3

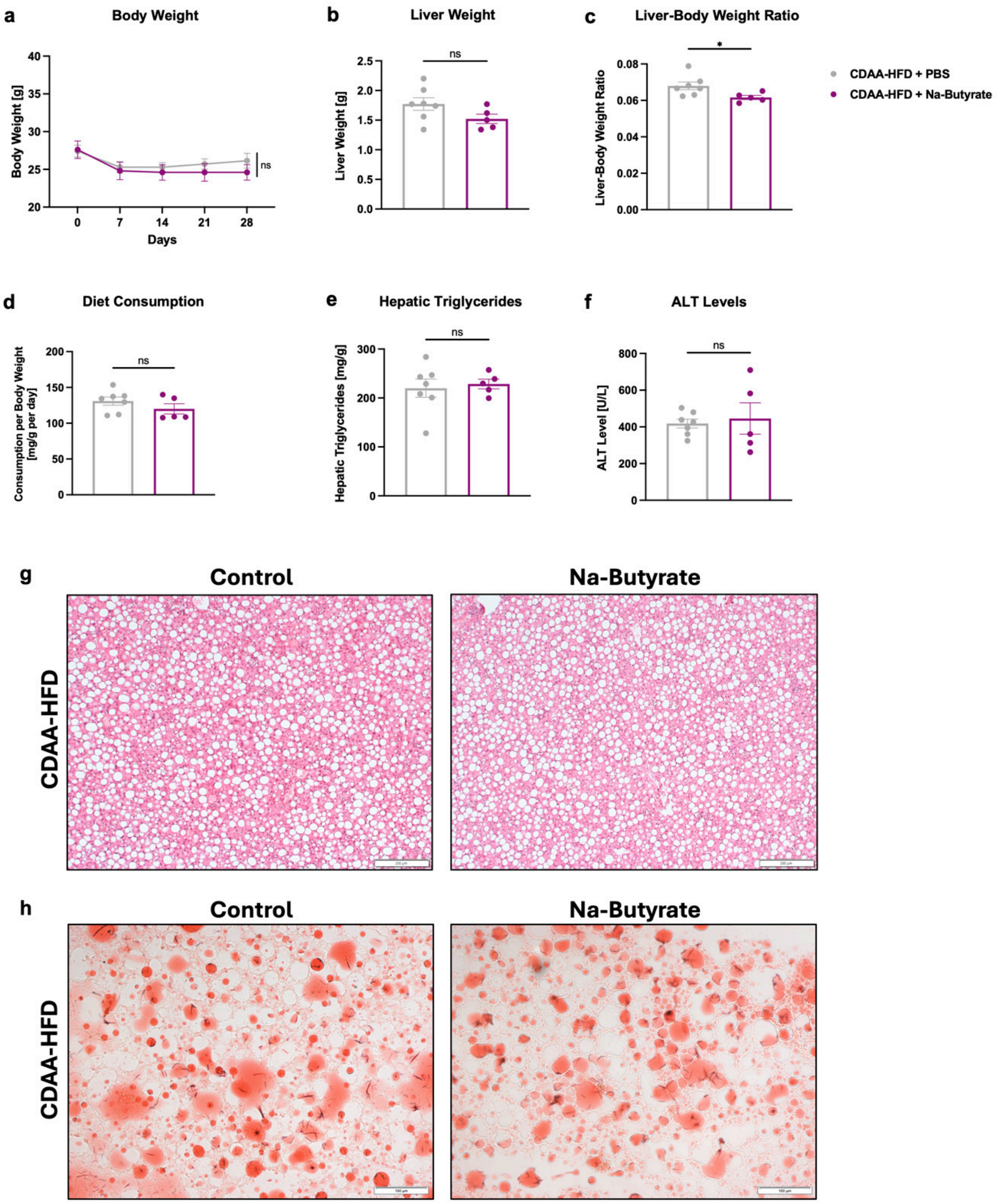

Figure S3. Limited effects of Sodium-butyrate on liver disease in CDAA-HFD-fed mice. C57BL/6 mice were placed on a CDAA-HFD (*n*=7-8) gavaged with sodium-butyrate

or PBS as a control for 4 weeks. **(a)** Absolute body weight. **(b)** Liver weight. **(c)** Liver weight-to-body weight ratio. **(d)** Daily food consumption in mg per g body weight per day. **(e)** Hepatic triglycerides. **(f)** Plasma ALT levels. **(g–h)** Representative liver sections after **(g)** hematoxylin and eosin staining (bar size=200 µm) and (**h**) after Oil Red O staining (bar size = 100 µm). Results are shown as mean ± s.e.m. p values were calculated using Mann-Whitney test **(a–f).** *p<0.05. ns indicates p > 0.05. *ALT,* alanine aminotransferase; *CDAA-HFD*, choline-deficient L-amino acid-defined high-fat diet; *PBS*, phosphate buffered saline.

Figure S4

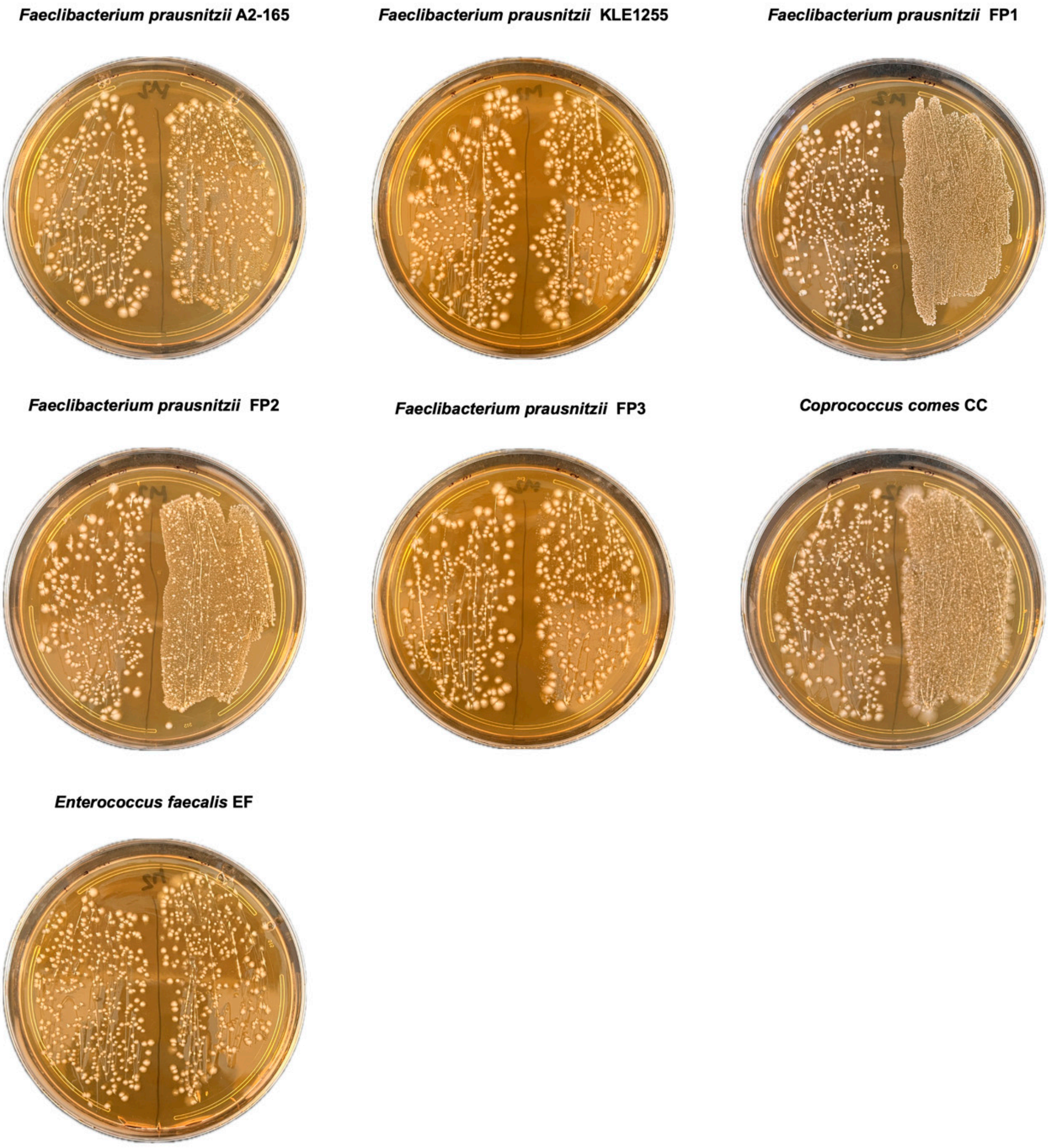

Figure S4. Appearance of butyrate-producing bacteria on M2GSC agar plates. 100µl of diluted (10^{-3} on the right and 10^{-6} on the left) bacterial culture media was plated on half of an M2GSC agar plate each and incubated in the anaerobic chamber for 72h.

Figure S5

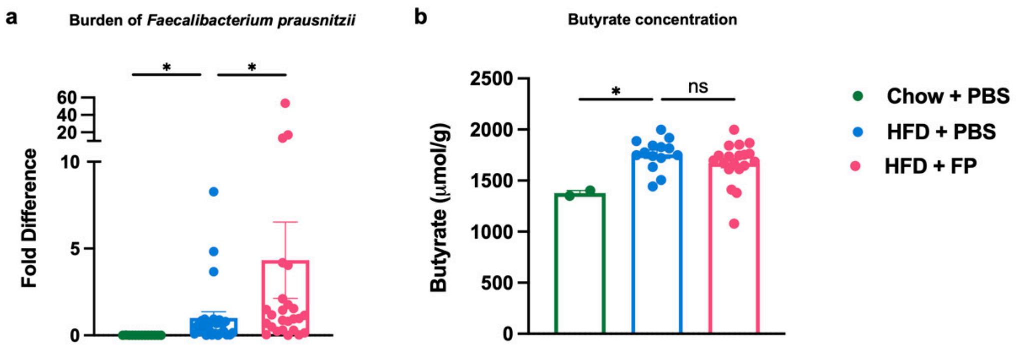

Figure S5. Orogastric gavage of *F. prausnitzii* increases abundance of *F. prausnitzii* in mouse feces. C57BL/6 mice were fed a chow diet (*n*=13) or HFD (*n*=25-26) with or without an isolated *F. prausnitzii* strain for 4 weeks**. (a)** Burden of *Faecalibacterium prausnitzii*. **(b)** Butyrate concentration. Results are shown as mean ± s.e.m. p values were calculated using Kruskal-Wallis test with Dunn's post hoc test. *p<0.05. ns indicates p > 0.05. *FP*, isolated *Faecalibacterium prausnitzii* strain; *HFD*, high fat diet; *PBS*, phosphate buffered saline**.**

Supplementary References

1. Torriani S, Zapparoli G, Dellaglio F. Use of PCR-based methods for rapid differentiation of Lactobacillus delbrueckii subsp. bulgaricus and L. delbrueckii subsp. lactis. Appl Environ Microbiol 1999;65(10):4351-6. (In eng). DOI: 10.1128/AEM.65.10.4351-4356.1999.
2. Maeda H, Fujimoto C, Haruki Y, et al. Quantitative real-time PCR using TaqMan and SYBR Green for Actinobacillus actinomycetemcomitans, Porphyromonas gingivalis, Prevotella intermedia, tetQ gene and total bacteria. FEMS Immunol Med Microbiol 2003;39(1):81-6. (In eng). DOI: 10.1016/S0928-8244(03)00224-4.
3. Hartmann P, Duan Y, Miyamoto Y, et al. Colesevelam ameliorates non-alcoholic steatohepatitis and obesity in mice. Hepatol Int 2022;16(2):359-370. (In eng). DOI: 10.1007/s12072-022-10296-w.
4. Roh YS, Zhang B, Loomba R, Seki E. TLR2 and TLR9 contribute to alcohol-mediated liver injury through induction of CXCL1 and neutrophil infiltration. Am J Physiol Gastrointest Liver Physiol 2015;309(1):G30-41. (In eng). DOI: 10.1152/ajpgi.00031.2015.
5. Niu M, Luo Z, Gong S, et al. Intestinal Epithelial Chemokine (C-C Motif) Ligand 7 Overexpression Enhances Acetaminophen-Induced Hepatotoxicity in Mice. Am J Pathol 2020;190(1):57-67. (In eng). DOI: 10.1016/j.ajpath.2019.09.009.
6. Fitzgerald CB, Shkoporov AN, Sutton TDS, et al. Comparative analysis of Faecalibacterium prausnitzii genomes shows a high level of genome plasticity and warrants separation into new species-level taxa. BMC Genomics 2018;19(1):931. (In eng). DOI: 10.1186/s12864-018-5313-6.
